# A Treatment Combination of IGF and EGF Promotes Hair Growth in the Angora Rabbit

**DOI:** 10.3390/genes12010024

**Published:** 2020-12-26

**Authors:** Bohao Zhao, Jiali Li, Qiuran Chen, Naisu Yang, Zhiyuan Bao, Shuaishuai Hu, Yang Chen, Xinsheng Wu

**Affiliations:** 1 College of Animal Science and Technology, Yangzhou University, Yangzhou 225009, China; 007623@yzu.edu.cn (B.Z.); li1193117036@163.com (J.L.); m15262241602@163.com (Q.C.); dx120180101@yzu.edu.cn (N.Y.); 18352764997@163.com (Z.B.); 18852726848@163.com (S.H.); yangc@yzu.edu.cn (Y.C.); 2 Joint International Research Laboratory of Agriculture & Agri-Product Safety, Yangzhou University, Yangzhou 225009, China

**Keywords:** hair follicle, IGF-1, EGF, gene regulation

## Abstract

The hair follicle (HF) growth cycle is a complex, multistep biological process, for which dysfunction affects hair-related diseases in humans and wool production in animals. In this study, a treatment combination of 10 ng/mL insulin-like growth factor-1 (IGF-1) and 20 ng/mL epidermal growth factor (EGF) significantly increased the elongation length of hair shafts for cultured HFs. The combined treatment of IGF-1 and EGF enhanced the proliferation of HFs and promoted HF growth and development in vitro. In vivo, the combined treatment of IGF-1 and EGF was subcutaneously injected into the dorsal skin in HF synchronized rabbits. The IGF-1 and EGF combination promoted the transition of the hair cycle from telogen to anagen and stimulated the growth of hair shafts. This IGF-1 and EGF combination maintained the structure of the HF and enhanced the cell proliferation of outer root sheaths and the dermal papilla within rabbit skin. The combined treatment of IGF-1 and EGF regulated HF-related genes, including *LEF1*, *CCND1* and *WNT2*, suggesting that IGF-1 and EGF play a positive role in HF growth and development. Utilization of the combined IGF-1 and EGF treatment may assist with hair and wool production and HF related diseases in mammals.

## 1. Introduction

The hair follicle (HF) is complex skin accessory organ, which has a unique structure and plays important roles in growth and development in mammals [1]. Under the regulation of endogenic and exogenous factors, HFs undergo a continuous and cycling process that consists of anagen (growing), catagen (involution), and telogen (resting) phases [2]. In order to better understand the molecular mechanisms that drive HF changes, HF-related cell lines and organ culture of isolated HFs have been utilized in vitro [3,4,5]. Furthermore, a number of animal models have been developed to examine the molecular regulation of HF by internal and external factors [6].

Many cell growth factors have been reported to be regulators of HF growth and development, including hepatocyte growth factor (HGF), keratinocyte growth factor (KGF), insulin-like growth factor-1 (IGF-1) and epidermal growth factor (EGF) [7,8,9,10]. Insulin-like growth factor 1 (IGF-1) is a key regulator of cell activity and biological processes [11,12]. Emerging evidence has demonstrated that IGF-1 affects the hair growth cycle, follicular proliferation and differentiation, and tissue remodeling [9]. IGF-1 promotes hair growth and plays an important role in the HF cycle and development in transgenic mice; with expression of IGF-1 correlated with androgenetic alopecia [13,14]. Furthermore, IGF-1 regulates the transition from anagen to catagen during the HF cycle [15].

Epidermal growth factor (EGF) also plays a critical role in the regulation of HF growth and development. In the outer root sheath (ORS), EGF and its receptor (EGFR) are downregulated in expression in HF growth [10]. EGF enhances cell proliferation of HF-derived mesenchymal stem cells through the EGFR/ERK and AKT signaling pathways [16]. In cultured, isolated human HFs, EGF stimulated elongation and maintained the growth of the HF [17]. EGF increased wool yields in Angora rabbits after subcutaneous injection and could induce regrowth of HFs [18].

In this study, we considered the role of IGF-1 and EGF in HF growth and development in Angora rabbits. A treatment combination of IGF-1 and EGF altered the growth of isolated HFs in culture and the cyclic regeneration of HFs in Angora rabbits. This growth factor combination also regulated the expression of HF growth and development related genes. These findings assist with an understanding of the biological functions of IGF-1 and EGF in HF growth and development and contribute to potential therapies for hair-related diseases in humans, and the improvement of wool production in animals.

## 2. Materials and Methods

### 2.1. Animals

HFs were isolated from male Angora rabbits. Thirty 7-month-old male Angora rabbits were used for the subcutaneous injection experiments. All rabbits were housed under the same conditions, including diet (feed pellet and grass), water and temperature. The experimental procedures in this study were approved by the Animal Care and Use Committee of Yangzhou University, China.

To determine the onset of the anagen phase and to estimate hair elongation for the Angora rabbits, the dorsal area of the experimental animals was removed by waxing and plucked. When HFs entered the anagen phase was determined by the appearance of light pink skin and by hair regrowth. The length of the hair shaft was measured and skin samples were collected. Samples were fixed in 4% formaldehyde, and paraffin sections were stained with hematoxylin–eosin (HE) for histological observations.

### 2.2. Hair Follicle Organ Culture

According to previous studies, the organ culture of whisker follicles is a more ideal model because of their superior size, prominent disposition, and rapid initiation of a new anagen phase compared to pelage follicles [19,20]. Organ culture was performed with whisker follicles isolated from Angora rabbits. Whisker pads were removed and whole whisker follicles were dissected under a stereo-microscope. Anagen whisker follicles were cultured in 24-well plates with Williams E Medium (Gibco, Paisley, Scotland, UK) in a humidified incubator at 37 °C and 5% CO_2_. The basal medium used was serum free. Whisker follicles were arranged into two groups: a treatment combination group (10 ng/mL IGF-1 and 20 ng/mL EGF) and a control group (not given any treatment). The culture medium was replaced with fresh medium every 2 days until day 6 post-treatment. The length of the hair shaft protruding from the HF was measured using a light microscope.

### 2.3. Hair Growth Model for Angora Rabbits

The 30 Angora rabbits were split into two groups: an IGF-1 and EGF treatment combination group and a vehicle-control group. For the treatment group, 2 ng IGF-1 and 4 ng EGF were dissolved in saline solution, then 200 μL of the mixed solution was subcutaneously injected into the dorsal skins of HF synchronized Angora rabbits. For the control group, 200 μL saline solution was subcutaneously injected into the dorsal skins of HF synchronized Angora rabbits. The solutions were injected each week until 21 days had elapsed. The growth and development of wools and skin and the onset of HFs were observed. Skin samples were collected at 0, 7, 14 and 21 days.

### 2.4. Immunofluorescence Staining

Rabbit skin tissues were fixed in 4% paraformaldehyde and embedded in paraffin. Embedded blocks were sectioned at 5 μm, then the paraffin sections were dewaxed and immersed in xylene Ⅰ and Ⅱ, and then sequentially in 100%, 95%, 85% and 75% alcohol for 5 min, and finally rinsed in deionized water. Antigen retrieval was processed in sodium citrate buffer, with samples washed three times in phosphate buffered saline (PBS) for 5 min after cooling to room temperature. Samples were then blocked and incubated with proliferating cell nuclear antigen (PCNA) mouse monoclonal antibody overnight at 4 °C. PCNA was used as this protein plays a key role in cell cycle regulation, cell division and cell proliferation [21]. Sections were washed in PBS three times and incubated with a goat anti-mouse IgG secondary antibody. 4’, 6-diamidino-2-phenylindole (DAPI) was applied for nuclear staining. The slides were then washed three times with PBS, and coverslips were mounted onto slides with anti-fading buffer mounting medium. The morphology of HFs was observed by fluorescence microscopy (OLYMPUS).

### 2.5. Quantitative Real-Time Polymerase Chain Reaction

Total RNA was isolated from skin using the RNAsimple Total RNA Kit (Tiangen, Beijing, China) and cDNA was obtained using HiScript II Q Select RT SuperMix for quantitative polymerase chain reaction (qPCR; Vazyme, Nanjing, China). Quantitative real-time polymerase chain reaction (qRT-PCR) was performed using AceQ qPCR SYBR^®^ Green Master Mix (Vazyme), according to the manufacturer’s instructions. Data were analyzed with QuantStudio^®^ 5. Relative gene expression levels were calculated using the 2^−ΔΔCt^ method [22] with gene expression normalized to glyceraldehyde 3-phosphate dehydrogenase (GAPDH). The specific primer sequences used for the qRT-PCR are listed in Table 1.

### 2.6. Statistical Analysis

All statistical analyses were performed using SPSS 22.0 (SPSS Inc., Chicago, IL, USA). Relative gene expression and hair shaft length were analyzed using a paired sample t-test. A minimum of three biological replicates were used for each analysis, and all error bars in the results represent the mean ± SD.

## 3. Results

### 3.1. The Treatment Combination of IGF-1 and EGF Promotes Hair Growth

**Figure 1:**
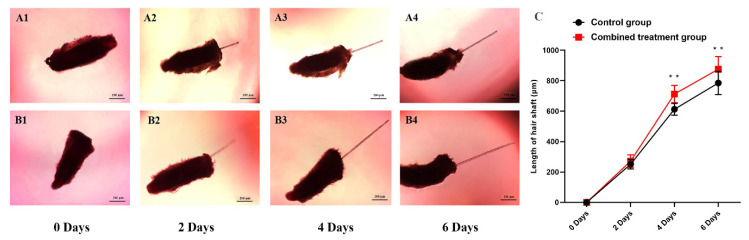
The combined treatment of IGF-1 and EGF regulates hair follicle growth. (**A1**–**A4**) Images of whisker follicles for the control group at 0, 2, 4 and 6 days. (**B1**–**B4**) Images of whisker follicles for the combined treatment group at 0, 2, 4 and 6 days. (**C**) The combined treatment of IGF-1 and EGF increased the elongation length of hair shafts. For significance, ** *p* < 0.01.

**Figure 2:**
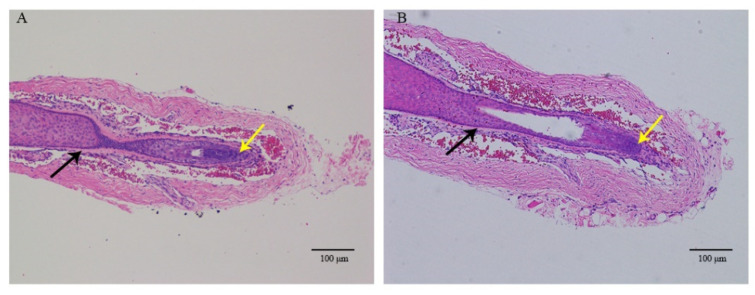
Hematoxylin–eosin (HE) staining of isolated hair follicles (HFs). (**A**) Control group (**B**) Combined treatment group. The black arrow points to the outer root sheath, and the yellow arrow labels the dermal papilla.

**Figure 3:**
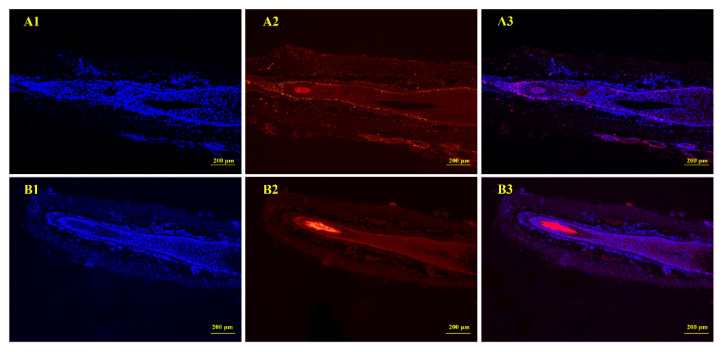
Immunofluorescence analysis of isolated HFs. (**A1**–**A3**) Control group (**B1**–**B3**) Combined treatment group. DAPI (nuclear) staining (blue) is indicated by 1, 2 indicates proliferating cell nuclear antigen (PCNA) staining (red), and 3 indicates the merged image.

Establishment of whisker follicles in organ culture was used to determine the influence of IGF-1 and EGF on hair growth. HFs maintained growing status during days 0 to 4, and there were still no changes in HF morphology after 4 days (Figure 1A,B). The treatment combination of IGF-1 and EGF significantly increased the elongation length of the hair shaft when compared with the control group at days 4 and 6 (Figure 1C). For the control group and combined treatment group, analysis of HE stains showed that there were no significant differences between the two groups. The structure of the HF was complete, the ORS was not atrophied, and the dermal papilla was clearly visible (Figure 2). Immunofluorescence analysis showed that PCNA was expressed at the bottom of the HF. The expression of PCNA was higher in the combined treatment than the control group, demonstrating that the treatment combination of IGF-1 and EGF enhanced the proliferative abilities of the HF and promoted HF growth in vitro (Figure 3).

### 3.2. IGF-1 and EGF Promoted Hair Growth in the Angora Rabbit

**Figure 4:**
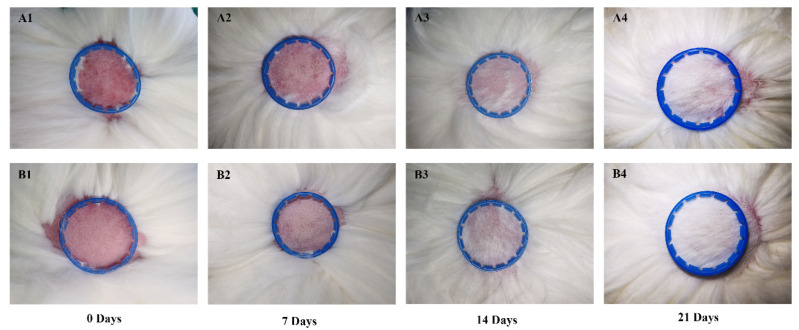
Morphological observation of rabbit skins. (**A1**–**A4**) Control group at 0, 7, 14 and 21 days. (**B1**–**B4**) Combined treatment group at 0, 7, 14 and 21 days.

To examine the response to growth factors in vivo, a HF synchronized model of Angora rabbits was established for which IGF-1 and EGF were subcutaneously injected into dorsal skin. From an observation of hair growth, the onset of hair growth was established at day 8 for the treatment combination of IGF-1 and EGF. By comparison, the onset of hair growth for the control group was at day 12. The experimental period was for 28 days, and the onset of anagen for the treatment group was sooner than the control group, suggesting that IGF-1 and EGF promoted hair growth and played a positive role in the transition from telogen into anagen during the hair cycle (Figure 4).

**Figure 5:**
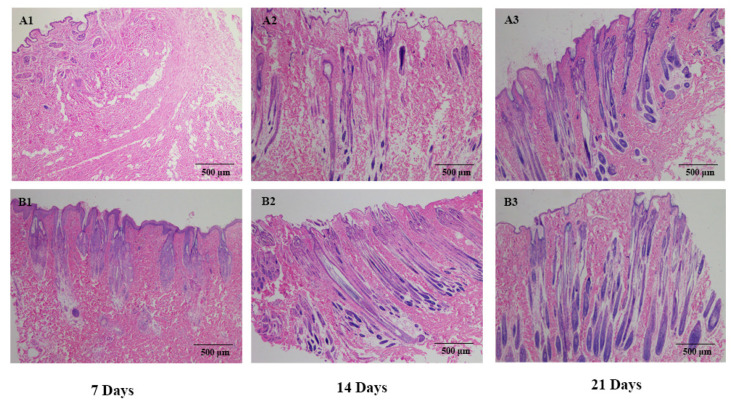
HE staining of sequential skin samples. (**A1**–**A3**) Control group at 7, 14 and 21 days. (**B1**–**B3**) Combined treatment group at 7, 14 and 21 days.

**Figure 6:**
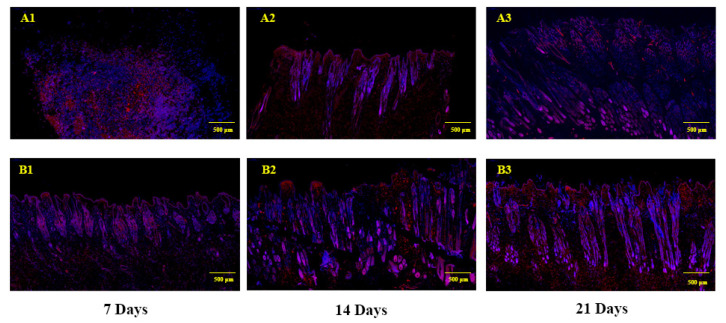
Immunofluorescence analysis of sequential skin samples. (**A1**–**A3**) Control group at 7, 14 and 21 days. (**B1**–**B3**) Combined treatment group at 7, 14 and 21 days. Red indicates PCNA staining, blue indicates DAPI (nuclear) staining.

Histological analysis showed that the structure of the HF began to recover at day 7, and that the HF for the treatment group was stronger than the control group. At day 14, the HF was completely into the anagen. The density and depth of the HF in the treatment group was higher than the control group. After 21 days of treatment, fur covered the entire dorsal skin, and there were no significant differences for the Angora rabbit between the control and treatment groups (Figure 5). The immunofluorescence of PCNA in rabbit skins showed higher expression in the treatment group than the control group. Furthermore, PCNA was highly expressed within the dermal papilla (DP) of the treatment group at day 14. At day 21, PCNA was highly expressed in the control group and treatment group. PCNA expression was localized to the DP in the control group, but PCNA was also highly expressed in the ORS and DP and with greater fluorescence observed for the treatment group (Figure 6).

### 3.3. IGF-1 and EGF Regulate Hair Follicle Development Related Genes in the Angora Rabbit

**Figure 7:**
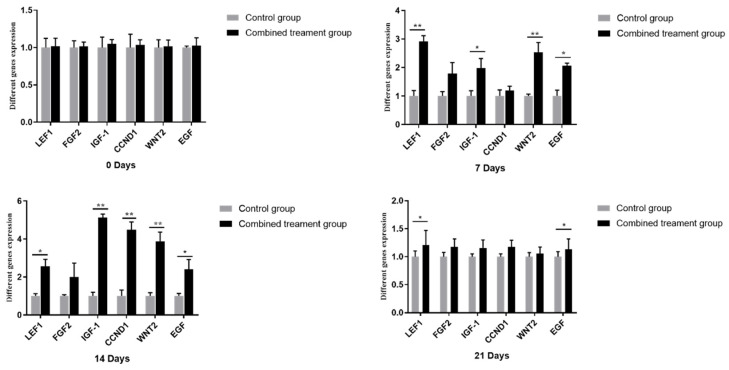
Examination of the influence of IGF-1 and EGF on HF development related genes at 0, 7, 14 and 21 days post-subcutaneous treatment. Histograms represent means ± SD of triplicate experiments. For significance, * *p* < 0.05, ** *p* < 0.01.

HF growth and development related genes were examined by qRT-PCR after treatment with the IGF-1 and EGF combination in HF synchronized rabbits. There were no differences between gene expression of the treatment and control groups at day 0. However, at day 7, mRNA expression of *LEF1*, *WNT2*, *IGF-1* and *EGF* were increased in the treatment group. At day 14, *LEF1*, *IGF-1*, *CCND1*, *WNT2* and *EGF* were significantly upregulated in the treatment group. At day 21 only the expression of the *LEF1* and *EGF* genes were higher than the control group. Collectively, the results indicate that the combination treatment of IGF-1 and EGF played a positive role in the regulation of HF growth and development (Figure 7).

## 4. Discussion

IGF-1 and EGF play important roles in the growth and development of the HF. In previous studies, it has been shown that IGF-1 is involved in cell apoptosis and promotes cell proliferation, and this has been evidenced within the dermal papilla [23]. In the absence of IGF-1, the anagen will enter the catagen stage during HF growth [24]. Furthermore, it has been shown that a knockdown of the IGF-1 receptor in mice will decrease the quantity of HFs and produce abnormal morphology of the HF [25]. EGF can maintain the renewal and integrity of the epidermal layer and affect the migration of stromal cells upwards away from the DP, but not towards the hair shaft with elongation of the outer root sheath [17,26]. In this study, a treatment combination of IGF-1 and EGF promoted the growth of isolated rabbit HFs cultured in vitro and positively influenced proliferation of HFs, as evidenced by immunofluorescent staining. Hence, collectively, the combination of the two growth factors promoted HF growth and development.

To further explore the role of the treatment combination, IGF-1 and EGF were subcutaneously injected into the dorsal skins of HF synchronized Angora rabbits. Previous studies have reported that IGF-1 can act as an anti-apoptotic factor and inhibit cell apoptosis in the anagen, and EGF can promote cell proliferation and induce the anagen in the HF cycle, as well as promote hair shaft growth [10,16]. For the in vivo experiments, hair shafts were first observed at day 8, after a combined treatment of IGF-1 and EGF. This was earlier than the control group, in which they appeared at day 10. Indeed, throughout the entire experiment period, the growth rate of the hair shaft for the control group was slower than the treatment group. In addition, the combination of IGF-1 and EGF promoted cell proliferation within the HF.

The Wnt/β-catenin signaling pathway plays an important role in skin and HF development [27]. As a key factor, Wnt2 can regulate the morphogenesis and development of the HF [28,29]. LEF1 is a downstream factor in the Wnt/β-catenin signaling pathway, and is expressed in the inner and outer root sheath cells, hair matrix cells and dermal papilla [30]. LEF1 is also highly expressed in the anagen, and can promote hair growth and development [31]. CCND1 acts as an anti-apoptotic factor, regulating the cell cycle and playing an important role in HF morphogenesis and the hair cycle [32,33]. The application of IGF-1 and EGF to the Angora rabbits upregulated mRNA expression of WNT2 and LEF1 from days 7 to days 14 after subcutaneous injection. The gene expression level of CCND1 was upregulated 14 days after treatment. Collectively, these results suggest that the combined treatment of IGF-1 and EGF play a positive role on HF growth and regeneration in Angora rabbits.

In conclusion, a combined treatment of IGF-1 and EGF promoted cell proliferation and accelerated hair growth in cultured and isolated HFs. Likewise, in vivo, the combined treatment of IGF-1 and EGF promoted hair growth and regeneration, increased the density of the HF, and influenced the expression of HF growth and development related genes. This study provides an insight into the potential use of a combined growth factor therapy for promoting hair growth; be it in humans or to improve wool production in animals.

## Figures and Tables

**Table 1:** Primer sequences used for quantitative real-time polymerase chain reaction (qRT-PCR).

Gene	Sequence (5′-3′)
*LEF1*	Forward primer: CATCTCGGGTGGATTCAGG
	Reverse primer: ATGAGGGATGCCAGTTGTG
*WNT2*	Forward primer: AGCCATCCAGGTCGTCATGAACCAG
	Reverse primer: TGCACACACGACCTGCTGTACCC
*FGF2*	Forward primer: GTGTGTGCAAACCGTTACCTT
	Reverse primer: TCGTTTCAGTGCCACATACCAG
*CCND1*	Forward primer: GAACGCTACCTTCCCCAGTGCTC
	Reverse primer: CCTCACAGACCTCCAGCATCCAG
*GAPDH*	Forward primer: CACCAGGGCTGCTTTTAACTCT
	Reverse primer: CTTCCCGTTCTCAGCCTTGACC
*IGF-1*	Forward primer: TTCAGAAGCAATGGGAAAAAT
	Reverse primer: TAGAAGAGATGCGAGGAGGAC
*EGF*	Forward primer: GCACAACACAGATGGAAGCAG
	Reverse primer: AGATACGGTCACCAAAAAGGG

## Data Availability

All data generated or analyzed during this study are available from the corresponding author on reasonable request.

## References

[1] Hardy M.H. (1992). The secret life of the hair follicle. Trends in Genetics.

[2] Alonso L., Fuchs E. (2006). The hair cycle. J. Cell Sci..

[3] Morris R.J., Liu Y., Marles L., Yang Z., Trempus C., Li S., Lin J.S., Sawicki J.A., Cotsarelis G. (2004). Capturing and profiling adult hair follicle stem cells. Nat. Biotechnol..

[4] Kwack M.H., Yang J.M., Won G.H., Kim M.K., Kim J.C., Sung Y.K. (2018). Establishment and characterization of five immortalized human scalp dermal papilla cell lines. Biochem. Biophys. Res. Commun..

[5] Kondo S., Hozumi Y., Aso K. (1990). Organ culture of human scalp hair follicles: Effect of testosterone and oestrogen on hair growth. Arch. Dermatol. Res..

[6] Orasan M.S., Roman I.I., Coneac A., Muresan A., Orasan R.I. (2016). Hair loss and regeneration performed on animal models. Clujul. Med..

[7] Lindner G., Menrad A., Gherardi E., Merlino G., Welker P., Handjiski B., Roloff B., Paus R. (2000). Involvement of hepatocyte growth factor/scatter factor and met receptor signaling in hair follicle morphogenesis and cycling. FASEB J..

[8] Guo L., Degenstein L., Fuchs E. (1996). Keratinocyte growth factor is required for hair development but not for wound healing. Genes Dev..

[9] Weger N., Schlake T. (2005). Igf-i signalling controls the hair growth cycle and the differentiation of hair shafts. J. Investig. Dermatol..

[10] Mak K.K., Chan S.Y. (2003). Epidermal growth factor as a biologic switch in hair growth cycle. J. Biol. Chem..

[11] Pollak M. (2008). Insulin and insulin-like growth factor signalling in neoplasia. Nat. Rev. Cancer.

[12] Key T.J., Appleby P.N., Reeves G.K., Roddam A.W. (2010). Insulin-like growth factor 1 (igf1), igf binding protein 3 (igfbp3), and breast cancer risk: Pooled individual data analysis of 17 prospective studies. Lancet Oncol..

[13] Li J., Yang Z., Li Z., Gu L., Wang Y., Sung C. (2014). Exogenous igf-1 promotes hair growth by stimulating cell proliferation and down regulating tgf-β1 in c57bl/6 mice in vivo. Growth Horm. IGF Res..

[14] Tang L., Bernardo O., Bolduc C., Lui H., Madani S., Shapiro J. (2003). The expression of insulin-like growth factor 1 in follicular dermal papillae correlates with therapeutic efficacy of finasteride in androgenetic alopecia. J. Am. Acad. Dermatol..

[15] Rudman S.M., Philpott M.P., Thomas G.A., Kealey T. (1997). The role of igf-i in human skin and its appendages: Morphogen as well as mitogen?. J. Investig. Dermatol..

[16] Bai T., Liu F., Zou F., Zhao G., Jiang Y., Liu L., Shi J., Hao D., Zhang Q., Zheng T. (2017). Epidermal growth factor induces proliferation of hair follicle-derived mesenchymal stem cells through epidermal growth factor receptor-mediated activation of erk and akt signaling pathways associated with upregulation of cyclin d1 and downregulation of p16. Stem. Cells Dev..

[17] Philpott M.P., Kealey T. (1994). Effects of egf on the morphology and patterns of DNA synthesis in isolated human hair follicles. J. Invest. Dermatol..

[18] Moore G., Thébault R.-G., Rougeot J., Van Dooren P., Bonnet M. (1987). Epidermal growth factor (egf) facilitates depilation of the angora rabbit. Ann. Zootech..

[19] Young R.D., Oliver R.F. (1976). Morphological changes associated with the growth cycle of vibrissal follicles in the rat. J. Embryol. Exp Morphol..

[20] Oshima H., Rochat A., Kedzia C., Kobayashi K., Barrandon Y. (2001). Morphogenesis and renewal of hair follicles from adult multipotent stem cells. Cell.

[21] Strzalka W., Ziemienowicz A. (2011). Proliferating cell nuclear antigen (pcna): A key factor in DNA replication and cell cycle regulation. Ann. Bot..

[22] Schmittgen T.D., Livak K.J. (2008). Analyzing real-time pcr data by the comparative c t method. Nat. Protoc..

[23] Itami S., Kurata S., Takayasu S. (1995). Androgen induction of follicular epithelial cell growth is mediated via insulin-like growth factor-i from dermal papilla cells. Biochem. Biophys. Res. Commun..

[24] Philpott M.P., Sanders D.A., Kealey T. (1994). Effects of insulin and insulin-like growth factors on cultured human hair follicles: Igf-i at physiologic concentrations is an important regulator of hair follicle growth in vitro. J. Invest. Dermatol..

[25] Liu J.P., Baker J., Perkins A.S., Robertson E.J., Efstratiadis A. (1993). Mice carrying null mutations of the genes encoding insulin-like growth factor i (igf-1) and type 1 igf receptor (igf1r). Cell.

[26] Lacouture M.E. (2006). Mechanisms of cutaneous toxicities to egfr inhibitors. Nat. Rev. Cancer.

[27] Andl T., Reddy S.T., Gaddapara T., Millar S.E. (2002). Wnt signals are required for the initiation of hair follicle development. Dev. Cell.

[28] Bayle J., Fitch J., Jacobsen K., Kumar R., Lafyatis R., Lemaire R. (2008). Increased expression of wnt2 and sfrp4 in tsk mouse skin: Role of wnt signaling in altered dermal fibrillin deposition and systemic sclerosis. J. Investig. Dermatol..

[29] Nie Y., Li S., Zheng X., Chen W., Li X., Liu Z., Hu Y., Qiao H., Qi Q., Pei Q. (2018). Transcriptome reveals long non-coding rnas and mrnas involved in primary wool follicle induction in carpet sheep fetal skin. Front. Physiol..

[30] Zhou P., Byrne C., Jacobs J., Fuchs E. (1995). Lymphoid enhancer factor 1 directs hair follicle patterning and epithelial cell fate. Genes Dev..

[31] Gat U., DasGupta R., Degenstein L., Fuchs E. (1998). De novo hair follicle morphogenesis and hair tumors in mice expressing a truncated β-catenin in skin. Cell.

[32] Cai C., Zhao G., Tian L., Liu L., Yan K., Ma Y., Ji Z., Li X., Han K., Gao J. (2012). Mir-15a and mir-16-1 downregulate ccnd1 and induce apoptosis and cell cycle arrest in osteosarcoma. Oncol. Rep..

[33] Xu X., Lyle S., Liu Y., Solky B., Cotsarelis G. (2003). Differential expression of cyclin d1 in the human hair follicle. Am. J. Pathol..

